# Network analysis highlights socio-demographic patterns of stone tool-using primates

**DOI:** 10.1038/s41598-026-61772-4

**Published:** 2026-08-01

**Authors:** Gwennan T. L. Giraud, Theo D. R. O’Malley, Jonathan S. Reeves, Amanda Tan, Sebastian Sosa, Suchinda Malaivijitnond, Lydia V. Luncz

**Affiliations:** 1 Lise Meitner Research Group Technological Primates, Max Planck Institute for Evolutionary Anthropology, 04103 Leipzig, Germany; 2 Center for the Advanced Study of Hominin Paleobiology, Department of Anthropology, George Washington University, Washington, DC, 20052 USA; 3 Interdisciplinary Center for Archaeology and the Evolution of Human Behavior, Universidade do Algarve, Campus Gambelas, 8005-139 Faro, Portugal; 4 Department of Anthropology, Durham University, Durham, DH1 3LE UK; 5 Department of Human Behavior, Ecology and Culture, Max Planck Institute for Evolutionary Anthropology, 04103 Leipzig, Germany; 6 Department of Biology, Faculty of Science, Chulalongkorn University, Bangkok, 10330 Thailand; 7 National Primate Research Center of Thailand, Chulalongkorn University, Saraburi, 18110 Thailand

**Keywords:** Tool user, Long-tailed macaques, Grooming network, Social position, Preferential associations, Ecology, Ecology, Evolution, Zoology

## Abstract

While the nutritional benefits and social learning of tool use in primates have been widely studied, far less is known about how tool use is associated with social life. We investigated the social aspects of tool use in a hybrid population of long-tailed macaques (*Macaca fascicularis aurea* × *M. f. fascicularis*) on Koram Island, Thailand, comprising both stone tool users and non-users. To assess potential socio-demographic patterns, we examined whether tool use differed between sexes and whether it was related to variation in dominance rank. Using social network analyses, we assessed whether tool users differed in their associations outside foraging contexts by analyzing their grooming interactions and positions within the grooming network. Males were more likely to use tools than females, whereas rank was unrelated to tool use. Tool users and non-users showed similar social connectivity, grooming rates, and numbers of grooming partners. However, grooming networks indicated that non-tool users preferentially associated with other non-users, whereas tool users showed no affiliative bias. By integrating individual characteristics with social network data, this study provides insight into how tool-use behavior integrates into primate social systems, particularly in populations with substantial variation in technological behavior.

**Supplementary Information:**

The online version contains supplementary material available at 10.1038/s41598-026-61772-4.

## Introduction

Once considered unique to humans, tool use is now known across multiple animal species^1–4^. The use of stone tools is of particular interest given its importance in human and hominin evolution. Yet, it remains exceedingly rare across the animal kingdom^5^. As a result, how stone tool use arises and the benefits it provides are a central focus across behavioral and evolutionary research^6,7^. The advantages of tool use have been largely investigated from a dietary and nutritional perspective, as tool use improves foraging efficiency through access to food resources that would otherwise remain inaccessible^8–14^. In some cases, stone tool use can also improve the quality of food that individuals can access^9^. Similarly, the factors that influence how tool use emerges, spreads, and persists tend to focus on food^15^. In contrast to clear nutritional benefits and environmental forces driving tool use, substantially less is known about how social dynamics and stone tool use are interlinked^16^.

Such investigations are important because social dynamics may be linked to both opportunities to acquire tool-use behaviors and the contexts in which these behaviors are expressed. In particular, the capacity to use tools is known to be acquired through social learning processes that require the opportunity to observe others using tools^17^. Remaining in proximity to tool users during foraging further facilitates such learning opportunities^18–20^. In non-human primates, evidence for this is seen in how young individuals bias their attention toward older, more proficient tool users, suggesting such learning opportunities are important to them^21–24^. Recent evidence further suggests that social tolerance can facilitate the cultural transmission of extractive foraging traditions in wild tool-using primates by increasing opportunities for social learning^25^. Similarly, individuals may benefit from remaining close to tool users during foraging, as tool-assisted processing can create scavenging opportunities for encased foods that non-tool users cannot otherwise access^22,23,26^.

Opportunities for social learning and close proximity are shaped by broader social dynamics. For instance, non-tool users have been shown to forage preferentially near lower-ranking tool users in long-tailed macaques (*Macaca fascicularis*), potentially reducing the risk of social conflict while maintaining access to these opportunities^23^. Socio-demographic factors may therefore be associated with tool-use behavior. Gaining access to learning and scavenging opportunities requires associating with tool users without the risk of conflict. Yet, in some primate species, strict despotic hierarchies limit tolerance toward lower-ranking individuals^27^. Rank, a key determinant of social status^28^, strongly shapes access to resources such as food^29^, social partners^30–36^, and reproductive opportunities^37^. In the context of stone tool use, higher-ranking individuals may therefore experience more opportunities for social contact with tool users or more stable access to tool use contexts, increasing their chances of becoming skilled tool users. Sex differences may also contribute to variation in tool-use behavior in primates. Male biases in tool use have been reported in several capuchin species, with males more likely to use tools than females^38–41^. A recent study on long-tailed macaques similarly found that tool use was primarily exhibited by males^42^. In this species, tool use does occur in both sexes^43^. However, sex differences have focused only on individuals who already use tools^43^, which leaves open the question of whether demographic characteristics such as sex or rank are associated with the likelihood of engaging in tool use in mixed populations containing both tool users and non-tool users.

Social relationships outside foraging contexts may also be associated with tool-use behavior. In primates, grooming is a key affiliative behavior for reinforcing social bonds and promoting tolerance within groups^44–47^. By maintaining or strengthening affiliative ties, grooming reduces social tension in competitive contexts, such as foraging^37,48,49^. Grooming interactions, therefore, provide a useful framework for examining how affiliative relationships are distributed within groups. Social network analysis offers a valuable framework to explore the relationship between stone tool use and social dynamics. These networks allow us to measure the structure and strength of relationships among individuals^50–53^. Social network analysis has increasingly been used to investigate the spread and social transmission of behavioral traditions, including tool use, in several animal species. For example, social networks have shown patterns of cultural transmission in tool-using dolphins^54^, direct social transmission of tool use in wild chimpanzees^55^, and social learning biases associated with novel foraging skills in vervet monkeys^56^. However, comparatively less attention has been given to whether individuals that differ in tool-use status also differ in their affiliative positions within networks. Comparing grooming relationships among tool users and non-tool users allows us to examine whether tool users differ in their social connectivity within the grooming network.

Patterns of social affiliation may additionally be structured according to similarities between individuals. Positive assortativity refers to the group-level tendency for individuals who share similar traits, behaviors, or social statuses to associate more frequently with one another^57,58^. This group-level pattern is also referred to as global homophily. In primates, various factors are known to contribute to homophilic grooming behaviors, such as kinship, sex, and hierarchical rank^59–61^. Whether similar patterns extend to tool-use status remains unknown. Although not testing for assortativity, previous research has reported strong foraging associations among tool users^26^. If grooming relationships are associated with tool-use status, non-random association patterns between individuals of similar status may appear. Therefore, individuals may preferentially groom others with a similar tool-use status. Conversely, the absence of assortativity at the group level does not necessarily imply that individuals lack social preferences altogether. Such local homophilic preferences may instead operate at a finer, subgroup-specific scale within the group.

In Thailand, the Koram Island population of long-tailed macaques provides a valuable opportunity to investigate these questions. This population contains both stone tool users and non-tool users coexisting within the same social group. Resident macaques are hybrids of both the habitual stone tool-using Burmese subspecies (*M. f. aurea*;^62–64^) and the common subspecies where stone tool use is absent or rare (*M. f. fascicularis*^42^). stone tools is correlated with the phenotype of *M. f. aurea*^26,65^. However, other factors are likely contributing to this pattern of tool use. Competency in tool use is related to ontogeny^66^, while social learning plays an important role in being a tool user^26^. The coexistence of tool users and non-tool users within a single interconnected social group therefore offers a rare opportunity to examine how demographic characteristics and affiliative social structure are associated with tool-use status within a natural primate population.

This study explores whether stone tool users differ from non-tool users in their socio-demographic characteristics and affiliative social integration within the Koram Island macaque population^67^. Given that tool use is embedded in the social system, we expect tool users and non-tool users to differ in their socio-demographic characteristics. Based on recent findings from other long-tailed macaque populations where tool use initially emerged among males^42^, we predict that males will be more likely to use stone tools than females. Considering the highly despotic nature of this species^27^, where social tolerance and proximity are strongly structured by dominance relationships, we also predict that higher-ranking individuals will be more likely to use tools than lower-ranking individuals. Second, opportunities for social learning and access to tool-mediated foraging contexts require repeated social contact with tool users^17–20^. We therefore expect tool use to be associated with patterns of affiliative social integration. In particular, we predict that social relationships established around tool use may extend beyond foraging contexts into the grooming domain. Consequently, stone tool users are expected to occupy more central positions within the grooming network compared to non-tool users. Finally, grooming relationships in primates are often non-random and structured by shared characteristics such as kinship, sex, and dominance rank^59–61^. We therefore investigate whether tool-use status similarly reflects shared social or behavioral contexts. We predict positive assortative grooming, with individuals preferentially grooming others sharing a similar tool-use status at both subgroup and group levels. Our results provide insight into the complex interplay between social factors and stone tool use within a population where tool users and non-tool users coexist, offering novel perspectives on the socio-demographic characteristics linked to stone tool use.

## Methods

### Study site and study group

Koram Island (N12° 14′ 32′′, E100° 0′ 34′′), in the Khao Sam Roi Yot National Park in Prachuap Khiri Khan Province of Thailand, is home to a population of 65–72 habituated long-tailed macaques (*M. fascicularis*). The island has a long rocky shore on which macaques use stone tools to crack open marine invertebrates exposed during the low tide. All resident macaques are hybrids of both the Burmese subspecies (*M. f. aurea*) and the common subspecies (*M. f. fascicularis*)^67^. However, only a subset of the population uses stone tools. Tourists regularly visit the site and provide food to the monkeys, and the animals are highly habituated to human presence.

### Behavioral data collection

Following Altmann’s methodology^68^, one researcher collected 5-min focal sampling data over 1109 h of observation across 227 days, from October 2013 to December 2014. Focal sampling was conducted mainly on the shore, where tool use behavior is prevalent and most visible. Individuals were sampled at random across low, medium, and high tides.

Age was defined using Tan’s methodology^66^ (infants < 1.5 years, juveniles 1.5–3.5 years, sub-adults > 3.5 years, and adults after first reproductive output). Out of the 72 individuals initially identified in the population, five (2 unidentified individuals, 1 juvenile, 1 sub-adult, and 1 adult) were excluded because no focal observation data could be collected for them. Eighteen infants were additionally excluded as they were too young to start using tools, and their social interactions were not independent of their mothers. Among the remaining individuals, seven (4 adults, 2 sub-adults, and 1 juvenile) lacked reliable phenotype classification based on hair patterns and were therefore excluded from the analyses, resulting in a final sample of 42 individuals (see Supplementary Table S1 online). Dominance rank and grooming network metrics were calculated prior to these exclusions using the broader observed social dataset, thereby reducing the potential influence of the final analytical subsampling on estimates of social network structure. The final sample included 13 juveniles, 9 sub-adults, and 20 adults. Among these 42 individuals, 27 were observed using tools (14 males, 13 females) and thereby defined as ‘tool users’, while 15 were defined as ‘non-tool users’ (4 males, 11 females; see Supplementary Table S2 online).

During each focal sampling event, agonistic and grooming interactions were recorded, including the direction of the behavior and the identity of the individuals involved. Hierarchical rank was determined from agonistic interactions according to the “youngest ascending principle”, where macaque infants inherit their rank from their mother, and the youngest is higher in rank compared to its siblings^27,69,70^. Hierarchical rank was calculated separately for each sex due to the distinct hierarchies observed for males and females in macaque societies^27,71–73^. To ensure comparable hierarchies between males and females, ranks were standardized across both sexes by dividing an individual’s rank by the total number of individuals of the corresponding sex in the sample and multiplying the result by ten. See Tan and colleagues^23^ for definitions of agonistic interactions. Grooming duration data were used for social network analysis (see below for social network construction).

### Matrix and social network building

Grooming interactions were used to construct a directed, weighted social network in which nodes represent individuals and ties represent grooming interactions. Tie direction corresponded to the direction of grooming behavior (received vs provided), whereas tie weights corresponded to the cumulative duration of grooming interactions between dyads across focal observations. Because individuals differed in focal observation time, grooming durations were standardized by individual observation effort prior to network metric calculation^50–52^ (see Supplementary Table S1 online for individual sampling effort). These standardized grooming rates were used as network weights.

The network position of each individual was characterized using three complementary social metrics: strength, degree, and eigenvector centrality^74^. These metrics capture distinct dimensions of social connectivity, including interaction intensity (i.e., tie weights), number of social partners (i.e., node connectivity), and broader embeddedness of individuals within the social network. We used these three metrics to compare the network positions of tool users and non-tool users. The strength quantifies the total duration of grooming interactions between a focal individual and its social partners^75^. The degree indicates the number of grooming partners with whom a focal individual interacted^76^. Both metrics can distinguish the direction of grooming interactions separately: the grooming provided (out-) and the grooming received (in-) by tool users and non-tool users. For example, in-strength refers to the total duration of grooming received by an individual. Both strength and degree metrics capture direct social relationships with immediate partners in the network. To complement these local measures, we used eigenvector centrality, which is calculated from an individual’s direct connections and the connections that their social partners have with other individuals^52,77–81^. Eigenvector centrality thus captures an individual’s broader embeddedness within the social network, beyond immediate neighbors. Resulting values range between 0 and 1, from the least centrally positioned individuals within the group to the most central.

Preferential grooming associations at the group-level were assessed using nominal assortativity, which measures the tendency of individuals to associate preferentially with others who share similar characteristics^57,58^. Assortativity values range from − 1 to 1, with positive values indicating homophily (preferential associations among similar individuals) and negative values indicating heterophily (preferential associations among dissimilar individuals). Values near 0 suggest no clear pattern of association.

To investigate whether preferential grooming associations might nonetheless operate at a finer, subgroup-specific scale (i.e., homophily within tool-use status categories), we additionally constructed ego networks centered on each individual’s tool-use status (tool user or non-tool user). For each focal individual, we separated grooming interactions with tool users from those with non-tool users (i.e., interaction type).

### Statistical analysis

All analyses were performed using R 4.2.1 software^82^. We used the *lme4*^83^ and *glmmTMB*^84,85^ packages accordingly. For all models, we checked for statistical assumptions with the *DHARMa* package^86^. To control for multicollinearity, we set a threshold of < 5 for the variance inflation factors ‘VIF’^87,88^ using the car and performance packages^89,90^. To check whether the selected full models were meaningful (i.e., different from random expectations), we assessed the joint significance of the tested models against null models using likelihood ratio tests (LRT). We then tested the effect of our main predictors by comparing each full model with a null model that excluded the test predictors and their interactions, but kept all other terms from the full model. Model stability was assessed by dropping one observation at a time. All models were stable except for the “Socio-demographic Model” with interaction between rank and age, where age varied widely (Supplementary Fig. S3 online). However, this was considered acceptable as the effect of this variable was recorded as non-significant and it was not a variable of primary interest. Moreover, parameter estimates were stable for all other variables.

#### Socio-demographic predictors of tool use (Socio-demographic Model)

Firstly, we assessed the influence of socio-demographic features on tool use behavior by using a binomial generalized linear model (GLM) with a logit link function to test if individuals were more likely to use tools (response variable) depending on their hierarchical rank and sex (predictors). Phenotype and age were included as control variables, as these are known to be associated with tool use behavior in this population of macaques^26,65,66^. Phenotype was determined based on the cheek-hair pattern of individuals and classified as either hybrid-like (resembling the Burmese phenotype) or common-like (resembling the Common phenotype). For details on the identification system, see Gumert and colleagues^65^. For this specific model, the control variable age had two levels: juveniles and sexually mature individuals. The latter group includes both adults and sub-adults. These age classes were grouped because ontogenetic studies indicate that tool users acquire their skills while they are still juveniles^66^. We included an interaction between age and rank to assess their combined association with tool use. This decision was based on evidence that age is often related to rank in macaque societies^91,92^. The effect of rank may therefore differ between adults and juveniles. To perform our analysis, we conducted full-reduced model comparisons, removing each variable one by one. An additional model was run without the interaction between age and rank.

#### Association between tool use behavior, phenotype, and social status (3 Grooming Models)

To investigate whether tool use extends beyond foraging contexts, we examined the association of tool use with social status and social position. First, to determine if using tools (predictor) affects an individual’s social position within its group during grooming, we set eigenvector centrality as the response variable in a model employing a gamma distribution with a logarithmic link (Social Position Model). Second, to specifically examine grooming interactions with direct social partners, we used two additional models to assess the influence of using tools (predictor) on the amount of grooming received (in-strength; response variable) and the number of grooming partners (in-degree; response variable) within the group. The model with the in-strength employed a Tweedie distribution with a logarithmic link function (Grooming Received Model), while the model with the in-degree used a Poisson distribution with a logarithmic link function (Grooming Partners Model). The Tweedie (truncated Gamma) distribution accommodates positive continuous data with a substantial proportion of zeros, making it particularly well-suited for our grooming duration variable^93^. Tweedie allows modeling both the count and the intensity components of interaction simultaneously, without needing to discard zero values from the dataset. To access this specific Tweedie distribution, we used the *glmmTMB* function.

The test predictors in the three models were tool-use status (tool user or non-tool user) and phenotype (Hybrid-like or Common-like). Phenotype was included in the grooming network analyses to account for potential genetic predispositions associated with hybrid status, which has been shown to influence tool use^26,65^. Rank and sex were included as control variables, as they are key factors influencing grooming exchanges^31,32,36,60,94–97^. Additionally, since young individuals significantly impact group social dynamics^32,98–102^, age was included as a control variable with three levels: juveniles, sub-adults, and adults. Sex is related to rank and differences in grooming interactions in macaque societies^27,71,94^. The effect of rank is therefore potentially different for males and females, and we also tested for the interaction between rank and sex to assess their joint influence on grooming inter-relations with tool users.

For those models, it was necessary to address that social data were determined from interaction data, leading to dependent observations among individuals. For instance, the time individual A spends grooming individual B is time that cannot be spent grooming individual C. This violates inferential statistical assumptions inherent in linear models using social matrices^103,104^. To comply with these assumptions, we thus permuted the social metric of each model (N = 10,000) using the *ANTs* R package^105^ before running the GLMs. For the model using the *glmmTMB function,* since this function is not compatible with the *ANTs* package, we performed the permutations manually in R using the *pbreplicate* function from the *pbapply* package^106^, which allowed repeated model fitting across permuted datasets. Significant p-values were obtained by comparing the distribution of the real data to that of the permuted data, which reflects what would be expected by chance.

#### Testing for preferential associations

##### Group-level patterns of grooming

Subsequently, to better understand the structure of social relationships in the context of tool use, we first tested for homophily patterns at the group level. Specifically, we assessed nominal assortativity^57,58,107^, applying the corrections proposed by Karimi & Oliveira^108^ to account for (1) the unequal group sizes of tool users (N = 27) and non-tool users (N = 15), and (2) potential asymmetries in relationships between categories (i.e., inter- and intra-group connectivity).

##### Subgroup-level patterns of grooming (Association Models)

Because other factors, such as sex, age, and hierarchical rank, can also shape grooming interactions^59–61^, we next examined whether homophilic tendencies might operate at a finer, subgroup-specific level. To do so, we compared the type of grooming interactions individuals formed with others using dyadic-level analyses. For each individual (tool user or non-tool user), we classified grooming interactions according to whether the partner was a tool user or a non-tool user. We specifically tested two aspects of these associations: the number of grooming partners (degree; Association Partners Model) and the strength of grooming interactions (Association Duration Model). To explore grooming dynamics in both tool-use statuses, we fitted two generalized linear mixed models (GLMMs) for each status, resulting in four models in total (two for each tool-use status). The first model is a zero-inflated Tweedie model with a logarithmic link function, using the grooming duration (strength) as the response variable (Association Duration Model). The second model is a zero-inflated Poisson model with a logarithmic link function, using the number of grooming partners (degree) as the response variable (Association Partners Model). These zero-inflated distributions help account for the moderate number of zeros in the dataset, which likely reflect structural absences of interaction rather than simple sampling gaps. While this approach does not directly test whether the absence of grooming depends on partner type, it allows us to focus on observed interactions. To access zero-inflated and Tweedie families, we used the *glmmTMB* function. 

Both models include the control variables rank, sex, and age class (juveniles, sub-adults, adults), along with the interaction between rank and sex. Because each individual appeared twice, once per interaction type, we included a random intercept for individual identity. To control for the imbalance between tool users (N = 27) and non-tool users (N = 15), we also added an offset term representing the size of each category. This offset helps us distinguish actual social preferences from differences in partner availability. Our main question was whether the tool-use status of the grooming partner affects the structure of an individual’s ego network. To test this, we permuted our test predictor, the partner’s tool-use status (i.e., interaction type), 10,000 times in each model. Because we did not permute the response variables, we did not interpret the effects of the control variables here. Our conclusions focused only on the effect of the tool-use status of the grooming partner (i.e., interaction type), assessed through this conditional permutation approach.

Overall, given the correlational nature of our analyses, we do not infer causal relationships between tool use and socio-demographic factors, but instead examine whether tool use is embedded within the broader social structure of the group.

## Results

### Socio-demographic predictors of tool use (Socio-demographic Model)

**Fig. 1:**
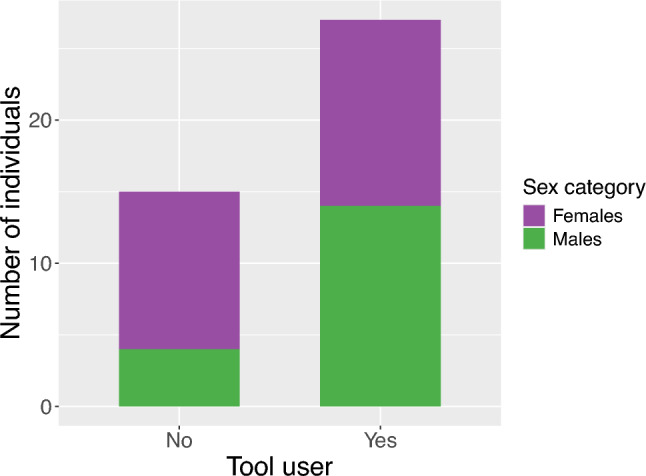
Males were more likely to use tools than females. Number of males (green) and females (purple) in the Koram Island group categorized by their likelihood to use tools.

Socio-demographic traits such as sex and phenotype were strong predictors of tool behavior in long-tailed macaques at Koram Island. Males were more likely to use tools than females, with 78% of males compared to 54% of females using tools (Fig. 1, full vs. reduced models: Sex X^2^ = 6.55, Df = 1, *p* < 0.05, Full model: Estimate 0.91 ± SE 0.74; Supplementary Tables S4 and S5 online for model outputs). Consistent with previous studies^26,65^, individuals with a hybrid-like phenotype were also more likely to use tools compared to those with a common-like phenotype, with 73% of hybrid-like individuals versus 50% of common-like individuals using tools (Phenotype X^2^ = 4.35, Df = 1, *p* < 0.05, Estimate 0.15 ± SE 0.71; Supplementary Fig. S6 online for the related figure). The interaction between hierarchical rank and age was not significant, indicating that in all age classes alike, hierarchical rank had no effect on tool usage (Rank:Age X^2^ = 1.03, Df = 1, *p* = 0.31; Supplementary Tables S4 and S5 online). After rerunning the model without the interaction, neither rank nor age was associated with the likelihood to use tools (Rank X^2^ = 0.84, Df = 1, *p* = 0.36; Age X^2^ = 1.80, Df = 1, *p* = 0.18, respectively).

### Association between tool use behavior, phenotype, and social status (3 Grooming models)

**Fig. 2:**
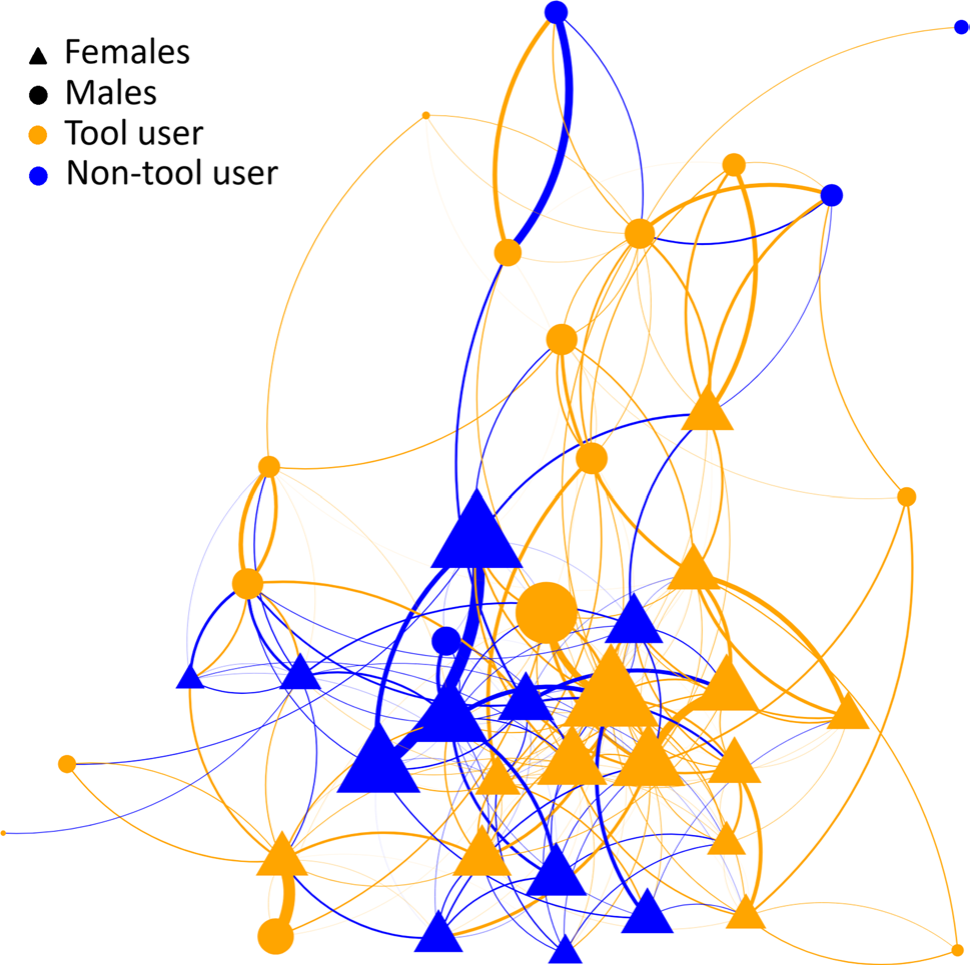
Social network of grooming interactions among Koram Island macaques: assessment of social position and social preferences within the group. This network illustrates grooming interactions among tool users (orange nodes) and non-tool users (blue nodes). Males are represented by circles and females by triangles. Node size reflects each individual’s eigenvector centrality, showing that tool users and non-tool users occupy comparable positions in the social network. Orange ties represent grooming given by a tool user, and blue ties represent grooming given by a non-tool user. Tie thickness corresponds to grooming strength between nodes. Visually, the grooming network provides no clear evidence of positive assortativity at the group level: individuals sharing similar tool-use status do not appear to structure the overall grooming network. However, dyadic analyses (interactions with tool users vs non-tool users), presented in the main text, revealed subgroup-level preferences: non-tool users preferentially engaged in grooming interactions with other non-tool users, whereas tool users did not show a comparable affiliative bias.

Our analysis did not provide evidence that tool users were more central in the grooming network than non-tool users. According to the permutation test, the results of the “Social Position Model” show that tool users had similar levels of social connectivity to group members compared to non-tool users (Fig. 2, Tool user *p* = 1; Supplementary Table S7 online for model output). The “Grooming Partners Model” similarly shows that both tool and non-tool users received grooming from a similar number of direct grooming partners (Tool user permutation test *p* = 0.51). The full-null model comparison for the “Grooming Received Model”, which assessed the duration of grooming received, was not statistically significant (full vs. null models: *X*^2^ = 8.97, Df = 7, *p* = 0.25). As a result, we were unable to draw a meaningful interpretation from this model.

Demographic traits did influence grooming connectivity. The “Grooming Partners Model” confirms that sex and hierarchical rank influenced grooming dynamics in this macaque population. Overall, hierarchical rank influenced grooming interactions differently in males and females (Grooming Partners Model, Rank:Sex Estimate 1.20 ± SE 1.06, permutation test *p* < 0.05; Supplementary Table S7 online for model output). Specifically, higher-ranked males were groomed by fewer partners than lower-ranked males. In contrast, higher-ranked females received grooming from more partners than lower-ranked females (Supplementary Fig. S8 online for the related figure). However, the grooming networks were not structured by genetic predispositions related to the hybrid-like phenotype (Social Position Model, Phenotype permutation test *p* = 0.12; Grooming Partners Model, Phenotype permutation test *p* = 0.61).

### Testing for preferential associations

#### Group-level patterns of grooming

Individuals’ tool-use status did not structure the grooming network (Fig. 2). The adjusted normalized mixing matrix showed that grooming ties were distributed both within and between categories: non-tool users groomed other non-tool users in 5.7% of all ties, tool users groomed other tool users in 22.1%, non-tool users groomed tool users in 12.6%, and tool users groomed non-tool users in 10.7% (Table 1). Although tool users interacted more frequently with each other than non-tool users did, the adjusted assortativity value was near zero (0.01), indicating that there was an absence of homophily at the group level: both tool users and non-tool users groomed individuals within and outside their category at comparable frequencies. Likewise, the symmetry-correction value (-0.01) showed no imbalance of connectivity between categories, further confirming that both groups were similarly connected within the grooming network.

**Table 1:** Adjusted mixing matrix for grooming interactions (corrected and normalized proportions) based on tool-use status (Tool users = TU; Non-tool users = NTU; arrow = direction of the grooming behavior).

	NTU → NTU	NTU → TU	TU → NTU	TU → TU
Adjusted mixing matrix	0.057	0.126	0.107	0.221

#### Subgroup-level patterns of grooming (Association Models)

Dyadic models of grooming ego networks revealed a clear asymmetrical, subgroup-specific bias. After controlling for sex, age, and rank, only non-tool users showed a significant preference: they groomed other non-tool users for longer bouts (Association Duration Model, Interaction type Estimate 0.33 ± SE 1.34, permutation test *p* < 0.0001; see Supplementary Table S9 online for full results) and had more grooming partners of the same tool-use status (Association Partners Model, Interaction type Estimate 0.28 ± SE 1.21, permutation test *p* < 0.0001). In other words, non-tool users clearly preferred each other over tool users. 

In contrast, tool users showed no selectivity with respect to partner tool-use status: they groomed both categories for similar amounts of time (Association Duration Model, Interaction type permutation test *p* = 0.18; see Supplementary Table S10 online for full results) and had comparable numbers of tool user and non-tool user partners (Association Partners Model, Interaction type permutation test *p* = 0.43).

## Discussion

As a rare but significant expression of technological behavior in animals, tool use has been widely studied in relation to ecological constraints and nutritional benefits^8,9,11,13,14^. Here, we examined whether and how stone tool use relates to social structure in a population of long-tailed macaques in which stone tool users and individuals who do not use stone tools coexist within the same social group. We specifically investigated this in a social context that reaches beyond foraging behavior. Across multiple analyses, we found that (i) males were more likely to use stone tools than females, (ii) tool use was not associated with higher rank, greater grooming centrality, or increased grooming received, (iii) tool use did not structure grooming relationships at the group-level; however, (iv) at a sub-group level, non-tool users preferentially groomed other non-tool users. This pattern was not found among tool users. Overall, these results suggest that tool use does not appear to structure grooming relationships in this group under the observed ecological conditions.

Our results show that males used tools more often than females. This male bias in stone tool use aligns with observations from another long-tailed macaque population that recently developed tool use during the COVID-19 pandemic, where the behavior was primarily exhibited by males^42^. Similar male biases have been reported in several capuchin species, where males were more likely to use tools than females^38–41^. By contrast, in a chimpanzee population where all individuals use tools, females crack nuts in trees more frequently than males, although this pattern may partly reflect a habituation bias^109,110^. Proposed explanations for this sex bias in tool use include differences in nutritional needs, physical capabilities, and social strategies for survival^38–41,43,109,111,112^. These species differences also parallel differences in dispersal systems. In chimpanzees, females are the dispersing sex, whereas in macaques and most capuchins, the dispersing sex is typically male^113–115^. Dispersal status may therefore be associated with variation in tool-use propensity across taxa. One possibility is that dispersing individuals, regardless of sex, may rely more strongly on flexible foraging strategies when leaving or entering novel social or unstable ecological environments. In meerkats (*Suricata suricatta*), dispersing individuals increase foraging activity, which may contribute to survival during dispersal periods^116^. Comparable data are currently lacking across primates. Nevertheless, dispersal-related differences in behavior between sexes may contribute to variation in tool-use propensity.

Our data further show that males, both tool and non-tool users, received grooming from fewer individuals than females (Supplementary Fig. S8 online). This result is broadly consistent with prior reports of sex differences in grooming behaviors, with males generally engaging less in grooming interactions than females^60,117–119^. Such sex differences in grooming are commonly linked to differences in dispersal and philopatry: males, as the dispersing sex, generally experience more unstable social relationships due to frequent migrations^113,120^, whereas females are philopatric and display strong matrilineal bonds, which they reinforce with grooming behaviors^60,97^. However, reduced grooming investment does not necessarily imply increased investment in tool use specifically, nor does it indicate how time is redistributed across other behavioral categories, such as foraging, movement, or other social activities. As such, while differences in social investment may be consistent with broader sex-specific strategies, the relationship between grooming interactions and tool-use propensity remains unresolved. The extent to which sex differences in tool use reflect social structure and dispersal patterns remains an open question that requires targeted longitudinal data.

Although these sex differences provide insight into variation in social investment, they do not explain whether tool use itself is associated with social integration. We therefore examined whether tool users occupied distinct positions within the group’s affiliative structure. Based on previous reports of strong associations among tool users during tool-assisted foraging^26^, we expected tool users to be more socially integrated. However, our results provide little support for this. Contrary to our expectations, tool users did not occupy more central positions in the grooming network, nor did they receive grooming from more partners than non-tool users. Furthermore, they did not hold higher ranks. Together, these results suggest that tool use is not associated with increased affiliative integration or elevated status in this population.

The absence of an association between rank and tool use suggests that tool users do not occupy higher-ranking positions than non-tool users in this population. This contrasts with our initial expectation that higher-ranking individuals might have more frequent access to tool-using contexts and more stable access to profitable foraging opportunities, thereby facilitating tool acquisition. In the present study system, ecological conditions likely reduce the opportunity for competition. Food availability was high during the study period, partly due to anthropogenic food provisioning, which reduced competition and the need for tool use to provide a foraging advantage. Shellfish and stones, the main targets of tool users, were also widely available and dispersed along the shore^25,65^, allowing monkeys to spread out. These abundant materials for tool-assisted foraging could not be monopolized, thereby limiting the extent to which access is constrained by dominance rank. As a result, rank-based differences in access to tool-related foraging opportunities may be weak or inconsistent, which is consistent with the absence of a detectable relationship between rank and tool use.

At the same time, this does not imply that rank is irrelevant to all aspects of foraging ecology. In a despotic social system such as that of long-tailed macaques^27^, rank can still structure tolerance and short-term competitive interactions, particularly in situations of local contest or interference. Indeed, even when overall resource abundance is high, individuals may still experience transient competition or displacement during feeding events, which can differentially affect low-ranking individuals. For instance, individuals of this population have been observed to scrounge opportunistically from tolerant, low-ranking tool users^23^. Feeding remains socially structured, with individuals often foraging in proximity along the shoreline. Tool users often exploit rapidly accessible resources such as oysters, whereas non-tool users may target alternative prey, such as crabs, that can require more search or handling time. These differences in foraging strategies may still generate occasional opportunities for food competition or tolerance-mediated access to desirable items without implying systematic reliance on tool users. At the same time, lower-ranking tool users are disadvantaged: despite their ability to access resources independently, they are often forced to stop foraging and even lose the benefits of their efforts when a higher-ranking individual steals their food. This suggests once again that the dominance relationship can still affect the realized benefits of foraging, even when tool use itself is not socially structured by rank. Even skilled foragers must navigate social challenges to fully capitalize on their abilities. Taken together, these findings suggest that the relationship between social rank and tool use is context-dependent and may be decoupled when ecological constraints on resource access are weak. Rather than reflecting a general absence of competitive dynamics, our results indicate that in this population, tool use is not embedded in a strongly rank-structured foraging system. Whether tool use becomes socially associated with rank may therefore depend on variation in ecological pressure, resource limitation, or contestability across contexts and time.

Our data also suggest that individuals did not selectively affiliate with tool users: neither tool users nor non-tool users showed a preference for grooming tool users. A parsimonious interpretation of this result is that tool-use status is not an important determinant of grooming relationships in this population. Under the ecological conditions of this study, tool use and grooming-based sociality operate largely as independent behavioral domains. Tool use is primarily embedded in foraging contexts, whereas grooming functions in the maintenance of social relationships^44^.

Our analyses of the preferential grooming associations showed two complementary but distinct results. At the group level, we found no evidence that individuals preferentially groom others based on shared tool-use status. However, a more fine-grained analysis revealed that non-tool users preferentially associated with one another during grooming interactions, whereas tool users did not exhibit a similar within-group preference. These results are not contradictory but instead indicate scale-dependent structure. The absence of global assortativity suggests that tool use does not organize grooming relationships across the group as a whole. The subgroup pattern among non-tool users indicates that heterogeneity exists within the non-tool user category that is not captured by group-level assortativity metrics. One possible explanation could be phenotype-based assortment, as tool-use status correlates with certain phenotypic traits^26,65^. Yet, these traits did not explain grooming interactions, suggesting that the observed affiliations are not simply a by-product of phenotype. Another possibility is that similarities in daily ranging or activity schedules contribute to increased interaction rates among non-tool users. At present, however, we cannot determine whether this pattern reflects active partner preference or differences in spatial overlap, activity budgets, or encounter rates among non-tool users. Instead, it reflects a measurable but currently unexplained tendency for non-tool users to associate with one another. Meanwhile, the broader and less selective grooming interactions of tool users prevent complete social segregation.

## Conclusion

Stone tool use in nonhuman primates offers a unique opportunity to examine the interplay between ecology, individual traits, and social structure. In macaques, such cultural behaviors are embedded in socio-ecological contexts^121,122^ and may be associated with sex-specific strategies and localized social interactions. Sex differences in tool use are evident, but their underlying causes remain unresolved and likely reflect multiple interacting ecological and social factors. Our results also indicate that stone tool use in this macaque population functions primarily as a foraging-related behavior rather than a social signal associated with grooming relationships. Tool use was not associated with higher rank, greater social centrality, or preferential affiliative integration. Grooming relationships were largely independent of tool-use status, with only limited subgroup-level structure observed among non-tool users. Overall, these results suggest that while tool use is embedded within a social system, it was not associated with the overall architecture of grooming relationships in this group or with differences in grooming-based social integration. More broadly, our findings emphasize that behavioral innovation and social organization can be partially decoupled. The relationship between them is likely contingent on ecological context and population social structure. From an evolutionary perspective, these results suggest that the maintenance of technological behaviors does not necessarily depend on enhanced social status or preferential affiliative relationships. Instead, behavioral innovations may persist through the interaction of ecological opportunity, individual propensity, and existing social structure, even when they do not become a major organizing feature of affiliative social networks.

## Supplementary Information

Supplementary Information.

## Data Availability

The datasets and analysis code supporting the findings of this study are publicly available in a GitHub repository: [https://github.com/GwennanGiraud/Socio-demographic-patterns-of-stone-tool-using-primates]. Any additional data is available from Dr. Lydia Luncz (lydia_luncz@eva.mpg.de), upon reasonable request.
