# Supplementary material for: Network analysis highlights socio-demographic patterns of stone tool-using primates
Source: Sci Rep. 2026 Aug 1;16:23720. doi: 10.1038/s41598-026-61772-4 (PMC13428744; doi:10.1038/s41598-026-61772-4)
Supplement: Supplementary file 1 — Supplementary Information. [file 41598_2026_61772_MOESM1_ESM.pdf]

Network analysis highlights socio-demographic patterns of stone tool-using primates

Gwennan T. L. Giraud[1,*], Theo D. R. O'Malley[1], Jonathan S. Reeves[1,2,3], Amanda Tan[4], Sebastian Sosa[5], Suchinda Malaivijitnond[6,7], and Lydia V. Luncz[1,2]

[1] Lise Meitner Research Group Technological Primates, Max Planck Institute for Evolutionary Anthropology, Leipzig, 04103, Germany
[2] Center for the Advanced Study of Hominin Paleobiology, Department of Anthropology, George Washington University, Washington, DC 20052, United States
[3] Interdisciplinary Center for Archaeology and the Evolution of Human Behavior, Campus Gambelas, Universidade do Algarve, Faro, 8005-139, Portugal
[4] Department of Anthropology, Durham University, Durham, DH1 3LE, United Kingdom
[5] Department of Human Behavior, Ecology and Culture, Max Planck Institute for Evolutionary Anthropology, Leipzig, 04103, Germany
[6] Department of Biology, Faculty of Science, Chulalongkorn University, Bangkok, 10330, Thailand
[7] National Primate Research Center of Thailand, Chulalongkorn University, Saraburi, 18110, Thailand

* Corresponding author: gwennan_giraud@eva.mpg.de, gwennangiraud@gmail.com

Supplementary information

Supplementary Table S1. Attribute table of all focal individuals (N=49) showing the age and sex class (M=male, F= female), rank, hair pattern phenotype, tool user status, number of focal collected, and sampling effort duration (minutes).

Subject name	Age class	Sex	Rank	Hair pattern	Tool user	Number of focal	Focal effort
Asurgai	Adult	M	5	NA	Yes	72	21600
Chang	Sub-adult	M	11	Hybrid	Yes	70	21000
Chohk	Juvenile	M	17	Common	Yes	75	22500
Chalaam	Adult	M	4	NA	Yes	66	19800
Chaihart	Sub-adult	F	22	NA	Yes	61	18407
Dam	Juvenile	F	18	Hybrid	No	71	21300
Dtao	Sub-adult	M	9	Common	Yes	69	20700
Fangnaam	Juvenile	M	20	Hybrid	Yes	70	21000
Fohn	Adult	F	14	Hybrid	No	151	45300
Grabain	Sub-adult	M	7	Common	Yes	49	14700
Heen	Sub-adult	M	8	Hybrid	Yes	79	23449
Hep	Adult	F	17	Hybrid	Yes	144	43239
Hoigap	Sub-adult	M	14	NA	Yes	68	20400
Hoikrang	Juvenile	F	20	Common	No	66	19730
Hoimalangpu	Juvenile	F	21	Hybrid	No	65	19500
Hoitak	Sub-adult	F	19	Hybrid	Yes	65	19500
Jan	Adult	F	8	Hybrid	Yes	71	21300
Jingrit	Juvenile	M	22	Common	No	73	21900
Jonesalat	Adult	M	15	Common	No	62	18600
Kaimook	Adult	F	2	Common	Yes	85	25500
Kamoy	Sub-adult	M	2	Common	Yes	59	17700
Kung	Juvenile	M	18	Hybrid	Yes	65	19500
Krabi	Adult	M	13	Hybrid	Yes	23	6900
Kangrang	Adult	M	10	Common	Yes	60	18000
Kwang	Adult	F	10	Hybrid	Yes	166	49800
Kwan	Sub-adult	M	3	Hybrid	Yes	45	13500
Lohm	Adult	F	7	Hybrid	Yes	69	20700
Mengaprun	Adult	M	12	NA	No	64	19200
Moo	Juvenile	M	23	Hybrid	Yes	73	21900
Mork	Adult	F	3	Hybrid	Yes	136	40800
Maintalay	Juvenile	F	13	Hybrid	No	67	20100
Naak	Juvenile	M	19	Common	Yes	73	21900
Neem	Adult	F	9	Hybrid	No	67	20100
Nguak	Adult	F	23	Common	No	72	21600
Nangrohm	Adult	F	25	Hybrid	Yes	124	37200
Nohk	Sub-adult	F	6	Common	Yes	187	56124
Nomsao	Adult	F	11	Hybrid	Yes	75	22500
Noi	Adult	F	24	Hybrid	Yes	67	20103
Narak	Juvenile	F	26	Common	No	73	21900
Pet	Adult	F	1	Hybrid	Yes	72	21600
Pla	Adult	F	15	NA	No	94	28200

Pladao	Juvenile	F	12	Hybrid	Yes	73	22245
Plawan	Sub-adult	M	1	Hybrid	Yes	62	18600
Poo	Adult	F	4	Common	No	89	26700
Priang	Juvenile	M	21	Hybrid	No	71	21300
Som	Juvenile	M	16	NA	No	67	20100
Suay	Adult	F	16	Common	No	67	20100
Yak	Adult	M	6	Common	No	61	18300
Ying	Adult	F	5	Hybrid	No	87	26100

Supplementary Table S2. Focal individuals (N=42) by their likelihood to use tools, phenotype, and demography.

	**Tool users**		**Non-tool users**	
	**Hybrid-like**	**Common-like**	**Hybrid-like**	**Common-like**
**Males**	8	6	1	3
**Females**	11	2	6	5
**Juveniles**	4	2	4	3
**Subadults**	5	4	0	0
**Adults**	10	2	3	5

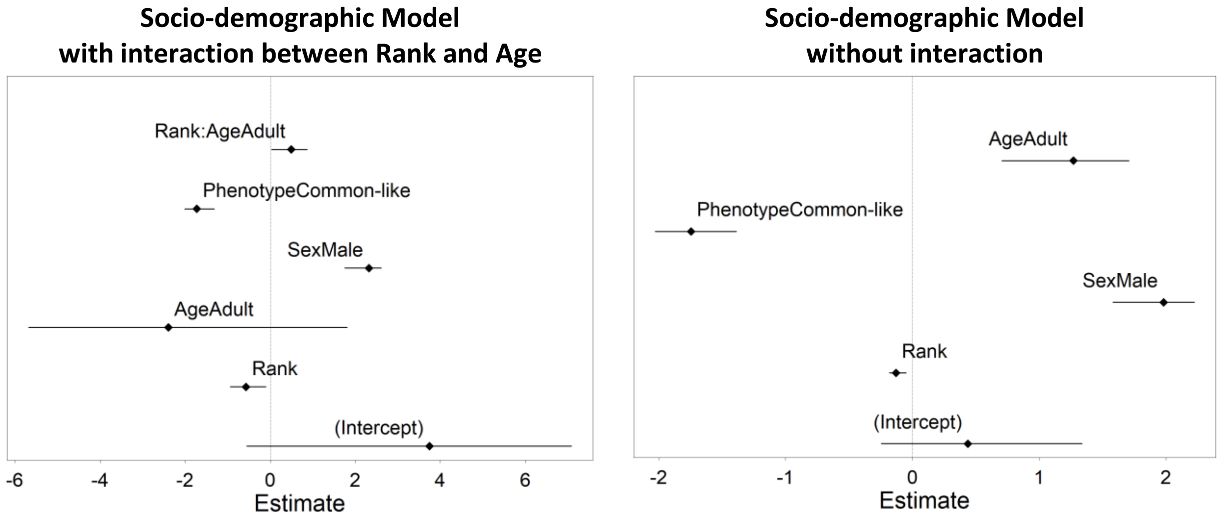

Supplementary Figure S3. Stability plot of the Socio-demographic Model, with Hierarchical Rank and Sex as the variables of interest, and Phenotype and Age as control variables. The model on the left includes an interaction between rank and age. The model on the right does not include the interaction.

Supplementary Table S4. Full-reduced model comparison investigating the likelihood of using tools in relation to individual features (Rank, Age, and Phenotype) for the Socio-demographic Model. Significant values are in bold. The results, both with and without the interaction between Rank and Age, are presented.

**Full model with interaction: Tool.user ~ Rank*Age + Sex + Phenotype, family = binomial (link="logit")**			
**Full model without (reduced model)**	**X^2**	**Df**	**P-values**
Rank	1.87	2	0.39
Age	2.82	2	0.24
Rank:Age	1.03	1	0.31
Sex	6.55	1	**<0.05**

Phenotype	4.35	1	**<0.05**
**Full model without interaction: Tool.user ~ Rank + Sex + Phenotype + Age, family = binomial (link="logit")**			
**Full model without (reduced model)**	**X^2**	**Df**	**P-values**
Rank	0.84	1	0.36
Sex	5.63	1	**<0.05**
Phenotype	4.72	1	**<0.05**
Age	1.80	1	0.18

Supplementary Table S5. Output of the Generalized Linear Model (Binomial regression with a logit link function) investigating the likelihood of using tools in relation to hierarchical rank and sex (Socio-demographic Model). The model also considered controlled predictors including age category and phenotype. For categorical variables, the reference levels are "Juvenile" for Age, "Female" for Sex, and "Hybrid-like" for Phenotype. Note that lower rank values indicate higher-ranking individuals. The results, both with and without the interaction between Rank and Age, are presented.

**Response variable**	**Fixed Effects**	**Estimate ± SE**	**Reverse logit Estimate ± SE**	**z Values**
Tool user (Socio-demographic Model with interaction)	Intercept	3.75 ± 3.70	0.977 ± 0.976	1.01
	Rank	-0.57 ± 0.48	0.36 ± 0.62	-1.19
	Age	-2.39 ± 3.86	0.08 ± 0.98	-0.62
	Sex	2.32 ± 1.05	0.91 ± 0.74	2.21
	Phenotype	-1.73 ± 0.88	0.15 ± 0.71	-1.96
	Rank:Age	0.49 ± 0.51	0.621 ± 0.624	0.98
Likelihood ratio test with null model: X^2=7.87, Df=3, p<0.05*				
Tool user (Socio-demographic Model without interaction)	Intercept	1.71 ± 0.93	0.85 ± 0.72	1.83
	Rank	-0.13 ± 0.14	0.47 ± 0.54	-0.91
	Sex	1.98 ± 0.92	0.88 ± 0.72	2.14
	Phenotype	-1.75 ± 0.86	0.15 ± 0.70	-2.03
	Age	-1.27 ± 0.96	0.22 ± 0.72	-1.32
Likelihood ratio test with null model: X^2=6.84, Df=2, p<0.05*				

* The null model excludes the test predictors (Rank and Sex) and the Rank–Age interaction, but retains all other terms included in the full model.

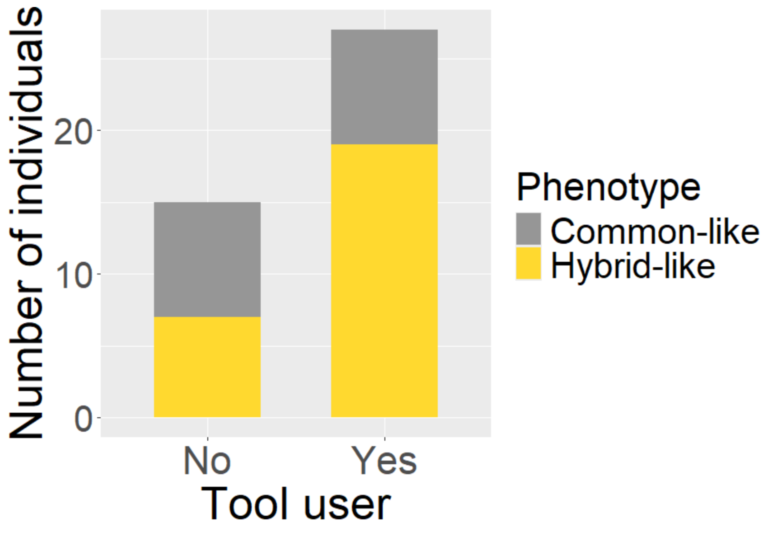

Supplementary Figure S6. Hybrid-like phenotypes are more likely to use tools. Number of common-like (gray) and hybrid-like (yellow) phenotypes in the Koram Island group categorized by their likelihood to use tools.

Supplementary Table S7. Output of the Generalized Linear Models (GLMs) investigating the social relationships of tool users within the grooming network and the potential influence of phenotype-associated genetic predispositions on grooming patterns (*Eigenvector centrality* (Social Position Model), Gamma regression with a logarithmic link function; *in-strength* (Grooming Received Model), Tweedie regression with a logarithmic link function; *in-degree* (Grooming Partners Model), Poisson regression with a logarithmic link function). All models also considered controlled predictors, including hierarchical rank, sex category, and age category. For categorical variables, the reference levels are “Non-tool user” for Tool-use status, “Hybrid” for Phenotype, “Female” for Sex, and “Juvenile” for Age. Note that lower rank values indicate higher-ranking individuals. Significant values are in bold.

Network	Response variable	Fixed Effects	Estimate ± SE	Reverse logarithmic functions Estimate ± SE	t/z Values	P-values
Exchange of grooming interactions	Group connectivity (*Eigenvector centrality*, Social Position Model)	Intercept	-0.14 ± 0.46	0.87 ± 1.59	-0.29	0.04
		Tool user	0.005 ± 0.27	1.01 ± 1.31	0.02	1
		Phenotype	-0.49 ± 0.24	0.61 ± 1.27	-2.04	0.12
		Rank	-0.07 ± 0.05	0.93 ± 1.05	-1.46	0.27
		Sex	-2.08 ± 0.58	0.13 ± 1.78	-3.60	**<0.01**
		Age Sub-adult	-0.08 ± 0.49	0.92 ± 1.64	-0.16	0.92
		Age Adult	-0.42 ± 0.32	0.66 ± 1.38	-1.31	0.29
		Rank:Sex	0.16 ± 0.09	1.18 ± 1.09	1.86	0.15
		Likelihood ratio test with null model: X^2=2.17, Df=2, p=0.11*				
Grooming received	Duration (*In-strength*, Grooming Received Model)	*Likelihood ratio test with null model**: X^2=8.97, Df=7, p=0.25*				
	Number of groomers (*In-degree*, Grooming Partners Model)	Intercept	2.00 ± 0.27	7.40 ± 1.31	7.43	0.65
		Tool user	0.17 ± 0.16	1.19 ± 1.17	1.09	0.51
		Phenotype	0.11 ± 0.14	1.11 ± 1.15	0.76	0.61
		Rank	-0.06 ± 0.02	0.94 ± 1.02	-2.42	0.18
		Sex	-1.76 ± 0.35	0.17 ± 1.42	-5.03	**<0.001**
		Age Sub-adult	0.58 ± 0.28	1.79 ± 1.32	2.11	0.20
		Age Adult	0.27 ± 0.20	1.31 ± 1.22	1.34	0.38
		Rank:Sex	0.18 ± 0.06	1.20 ± 1.06	3.24	**<0.05**
		Likelihood ratio test with null model: X^2=1.38, Df=2, p=50*				

* The null model excludes the test predictors (Tool user and Phenotype), but retains all other terms included in the full model.
** The null model excludes all predictors.

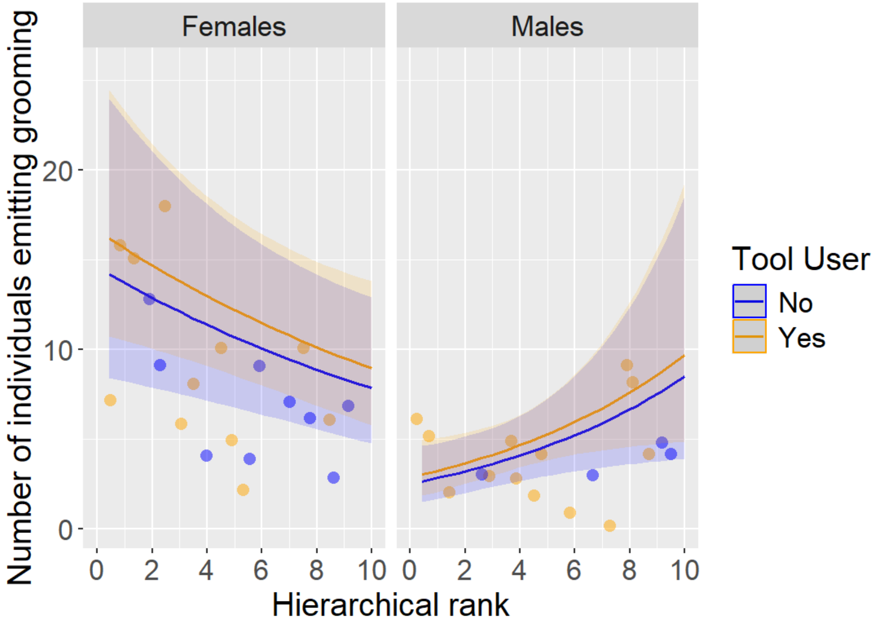

Supplementary Figure S8. Grooming Partners Model: Rank had an opposite effect on females and males. Predicted probabilities (with median and confidence intervals) of the number of individuals emitting grooming toward tool users (orange) or non-tool users (blue) according to sex category and hierarchical rank (standardized). Lower rank values indicate higher-ranked individuals.

Supplementary Table S9. Full results of Association Duration and Association Partners Models, showing the effect of **interaction type (grooming with a tool user *vs.* non-tool user) on grooming strength and number of partners of non-tool users.** The Association Duration Model is a zero-inflated Tweedie mixed model with a logarithmic link function. The Association Partners Model is a zero-inflated Poisson mixed model, also with a logarithmic link function. For categorical variables, the reference levels are “Non-tool user” for Interaction type, “Female” for Sex, and “Juvenile” for Age. Note that there were no subadults among non-tool users. Lower rank values indicate higher-ranking individuals.

Response variable based on **non-tool users**’ ego networks	Fixed Effects	Estimate ± SE	Reverse logarithmic function Estimate ± SE	z Values	P-values[1]
Association Duration Model (Strength)	Intercept	-5.84 ± 0.62	0.003 ± 1.86	-9.40	
	Interaction type	-1.10 ± 0.29	0.33 ± 1.34	-3.73	<0.0001
	Rank	-0.06 ± 0.07	0.94 ± 1.07	-0.91	
	Sex	-2.90 ± 1.40	0.05 ± 4.07	-2.07	
	Age Adult	0.20 ± 0.40	1.22 ± 1.49	0.51	
	Rank:Sex	0.30 ± 0.18	1.34 ± 1.19	1.69	
	Likelihood ratio test with null model: X^2=11.59, Df=1, p<0.001*				
Association Partners Model (Degree)	Intercept	-0.35 ± 0.38	0.70 ± 1.46	-0.94	
	Interaction type	-1.28 ± 0.20	0.28 ± 1.21	-6.56	<0.0001
	Rank	-0.06 ± 0.04	0.94 ± 1.04	-1.43	

	Sex	-3.72 ± 1.12	0.02 ± 3.06	-3.32	
	Age Adult	0.243 ± 0.240	1.28 ± 1.27	1.01	
	Rank:Sex	0.38 ± 0.13	1.47 ± 1.14	2.86	
Likelihood ratio test with null model: X^2=53.10, Df=1, p<0.0001*					

* The null model excludes the test predictors (Interaction type), but retains all other terms included in the full model.
[1]Empiric p-values were obtained by permuting only the "Interaction type" variable 10,000 times, following a conditional permutation approach. Other covariates (Rank, Sex, Age) were included as controls but were not tested formally. Their p-values are not reported and should not be used for inference.

Supplementary Table S10. Full results of Association Duration and Association Partners Models, showing the effect of **interaction type (grooming with a tool user *vs.* non-tool user) on grooming strength and number of partners of tool users.** The Association Duration Model is a zero-inflated Tweedie mixed model with a logarithmic link function. The Association Partners Model is a zero-inflated Poisson mixed model, also with a logarithmic link function. For categorical variables, the reference levels are "Non-tool user" for Interaction type, "Female" for Sex, and "Juvenile" for Age. Note that lower rank values indicate higher-ranking individuals.

Response variable based on **tool users**' ego networks	Fixed Effects	Estimate ± SE	Reverse logarithmic function Estimate ± SE	z Values	P-values[1]
Association Duration Model (Strength)	Intercept	-6.09 ± 0.47	0.002 ± 1.60	-12.94	
	Interaction type	0.24 ± 0.19	1.27 ± 1.20	1.29	0.18
	Rank	-0.06 ± 0.04	0.94 ± 1.04	-1.52	
	Sex	-1.24 ± 0.53	0.29 ± 1.70	-2.35	
	Age Sub-adult	-0.04 ± 0.48	0.96 ± 1.61	-0.09	
	Age Adult	-0.27 ± 0.43	0.76 ± 1.53	-0.63	
	Rank:Sex	0.08 ± 0.09	1.09 ± 1.10	0.88	
Likelihood ratio test with null model: X^2=2.40, Df=2, p=0.30*					
Association Partners Model (Degree)	Intercept	-0.72 ± 0.38	0.49 ± 1.47	-1.87	
	Interaction type	0.09 ± 0.12	1.09 ± 1.13	0.72	0.43
	Rank	-0.07 ± 0.03	0.93 ± 1.03	-2.23	
	Sex	-1.78 ± 0.42	0.17 ± 1.52	-4.27	
	Age Sub-adult	0.34 ± 0.38	1.41 ± 1.47	0.89	
	Age Adult	-0.01 ± 0.36	0.99 ± 1.43	-0.02	
	Rank:Sex	0.18 ± 0.08	1.20 ± 1.08	2.30	
Likelihood ratio test with null model: X^2=5.29, Df=2, p=0.07*					

* The null model excludes the test predictors (Interaction type), but retains all other terms included in the full model.
[1]Empiric p-values were obtained by permuting only the "Interaction type" variable 10,000 times, following a conditional permutation approach. Other covariates (Rank, Sex, Age) were included as controls but were not tested formally. Their p-values are not reported and should not be used for inference.
